# Peroxisome Proliferator-activated Receptor (PPAR)-γ Modifies Aβ Neurotoxin-induced Electrophysiological Alterations in Rat Primary Cultured Hippocampal Neurons

**DOI:** 10.22037/ijpr.2019.1100783

**Published:** 2019

**Authors:** Farideh Bahrami, Alireza Asgari, Narges Hosseinmardi, Mahyar Janahmadi

**Affiliations:** a *Neuroscience Research Center, Department of Physiology, Faculty of Medicine, Baqiyatallah University of Medical Sciences, Tehran, Iran.*; b *Neuroscience Research Center, Department of Physiology, School of Medical, Shahid Beheshti University of Medical Sciences, Tehran, Iran.*

**Keywords:** Alzheimer’s disease, Neurodegenerative diseases, Ca^2+^ channel current, PPAR-γ, Amyloid Beta (Aβ), Hippocampal pyramidal neurons

## Abstract

Alzheimer’s disease (AD) is undoubtedly one of the serious and growing public health challenges in the world today. There is an unmet need for new and effective preventative and therapeutic treatment approaches for AD, particularly at early stages of the disease. However, the underlying mechanism against Aβ-induced electrophysiological alteration in cultured hippocampal pyramidal neurons is still not fully understood. This study investigated the impacts of activation and inhibition of PPAR-γ/δ on the Aβ-induced functional toxicity, which occured before cell death, using patch clamp technique. Findings demonstrated that Aβ treatment alone altered the normal electrophysiological properties and reduced the Ca^2+^ channel currents in primary cultured hippocampal pyramidal neurons without any major changes either in cell structure, as evidenced by electron microscope examination, or cell viability. Rosiglitazone (30 µM), a potent PPAR-γ activator, when co-treated with Aβ (100 nM) prevented almost completely the induction of function toxicity of Aβ, as evidentiated by restored normal appearing electrophysiological properties. Inhibition of PPAR- γ/δ by FH535 (15 µM), an inhibitor of both Wnt/beta-catenin signaling and PPAR- γ and δ activity, when applied in combination of Aβ not only worsen the toxic electrophysiological effects of Aβ on firing frequency, membrane resistance and cell viability, but also even preserved the suppressive effect of Aβ on Ca^2+^ channel current when compared to control condition. Overall, these findings suggest that PPAR-γ activation could be a potential candidate to prevent the functional changes induced by low concentration of Aβ which may possibly occur in neurons during early stages of AD.

## Introduction

Alzheimer’s disease (AD) is a progressive neurological disorder, which is one of the most common causes of dementia in old age and is undoubtedly one of the serious and growing public health challenges in the world today (1). In AD, underlying mechanisms are multiple and complex which include Ca^2+^ homeostasis dysregulation, accumulation of amyloid beta (Aβ) neurotoxin, and hyperphosphorylation of tau protein. However, it is proposed that accumulation of Aβ may be the primary cause of AD pathology and a tau hyperphosphorylation is a downward event induced by an imbalance of Aβ production and clearance (2). The Aβ peptide plays an important role also in neuronal dysfunction in AD (3). On the other hand, AD has been reported to be associated with inflammation, lipid dyshomeostasis, and regional dysregulation of glucose metabolism within the brain (4-6). In this context, it has also been shown that the peroxisome proliferator activated receptor-γ (PPARγ), which is a prototypical ligand-activated nuclear receptor play an important role in coordination of lipid, glucose, and energy metabolism and agonists of this receptor have been found to be effective in the reduction of inflammation and memory impairment associated with AD (7, 8). There is an experimental evidence indicating that rosiglitazone, an agonist of PPARγ, reduces accumulation of Aβ1-42 in Alzheimer′s transgenic mice and attenuates learning and memory deficits in TG2576 mice (9). It has also been shown that rosiglitazone can improve spatial memory performance possibly by a decrease in the insoluble Aβ (1-42) in APP/PS1 mice (10). In addition, experimental evidence demonstrated that rosiglitazone improves synapse formation and plasticity and prevents the Aβ-induced LTP deficit in hippocampal slices (11). However, although there is also a close relationship between Aβ neurotoxicity, loss of Wnt signaling and PPARγ function in the pathogenesis of several neurological disorders, including AD, Miller *et al.* (2011) by reviewing some clinical studies suggested that rosiglitazone, a PPARγ agonist, should not be used as a therapy for AD, possibly at middle stages of AD (12-16).

The Wnt signaling pathway has been extensively studied for its different roles in neural development and synaptic growth (17, 20). It has been shown that Wnt ligands and receptors are expressed in different areas of the brain that are involved in synaptic plasticity (18, 19). The Wnt ligands regulate changes in neuronal cell shape and pre- and postsynaptic terminals, which thereby may contribute to the synaptic function and learning, therefore, they would appear that play modulatory role in synaptic function (19, 21 and 22). Despite studies demonstrateing that rosiglitazone activates Wnt signaling, which could be beneficial in AD treatment, there is, however, a little evidence of its detailed electrophysiological consequences against low dose of Aβ. 

Amyloid-β has been shown to bind to the several members of the Frizzled family of Wnt receptors and thereby switches off the canonical Wnt/β-catenin signaling pathway and blocks the downstream signaling cascade (3, 23). The suppressive effect of Aβ on β-catenin has been also shown in cultured neurons, which thereby interferes with normal Wnt signaling (23, 24). Hayashi *et al.* (2009) have suggested that the activity-dependent Wnt release as a trophic agent modulates neuronal excitability (25); therefore, inhibition of Wnt signaling may affect neuronal electrical function. In the present study, the impacts of co-application of either rosiglitazone, as a PPAR-γ activator, or FH535, which is a dual inhibitor of β-catenin pathway and the PPAR-γ/δ and Aβ on excitability and electrophysiological properties of rat hippocampal cultured pyramidal neurons were examined (26, 27). In addition, the possible involvement of voltage-gated Ca^2+^ channel on the effects of co-treatment of either rosiglitazone and Aβ or FH535 and Aβ on electrophysiological characteristics of cultured pyramidal neurons was also investigated. 

## Experimental

*Brain dissection and cellculture*

All experimental procedures were approved by the institutional Animal Care and ethical committee of Shahid Beheshti University of Medical Sciences.

Primary hippocampal cell cultures were prepared from 80 brains of 2-4 day-old (P2-P4) Wistar rats according to the method described by Brewer *et al.* (28). Twenty replicates were done (four replicates for each experimental group) and in each replicate four rat pups were used. Briefly, pups were decapitated after being anesthetized on ice, and hippocampi were dissected into ice-cold dissociation buffer containing calcium and magnesium free Hank’sbalanced salt solution (0.976%), sodium bicarbonate (0.035%, Sigma, UK) and pyruvate (1 mM), HEPES(10 mM) with a pH 7.4. Then, the tissue was gently triturated (15-18 times) through a fire-polished Pasteur pipette until it was dispersed into a homogenous suspension. The dispersed cells were centrifuged for 5 min at 1000 rpm, Neurons were then cultured on glass coverslips (16 mm in diameter) pre-coated with poly-L-lysine (0.01% poly-L-lysine ,Sigma UK) in a B27/neurobasal medium containing B27 (2%), neurobasal (96.75%), and L-glutamine (0.5 mM, Sigma, UK without antibiotic at a density 1-2 × 10^5^ cell/cm^2^. Antibiotics were not applied to the nutrient medium because they could affect the spontaneous activity (29). The cells were incubated at 37 °C under 5% CO_2_ and fed twice weekly. The morphological changes and growth of the neurons were observed using an Olympus IX71 inverted microscope (Olympus, Japan). The electrophysiological properties of cultured hippocampal neurons were recorded at day 14 *in-vitro* (DIV-14) using whole-cell patch-clamp method, under current and voltage clamp conditions.

*Whole-cell patchclamp of cultured hippocampal neurons*

**Figure 1:**
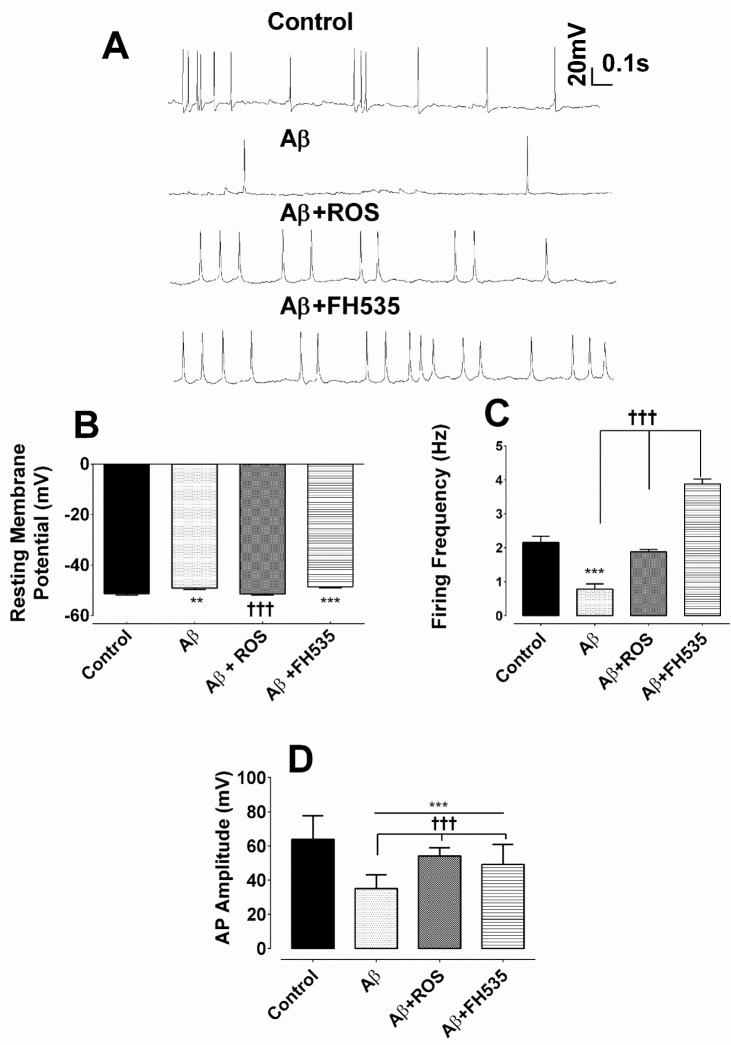
Alterations of resting membrane potential and firing responses following exposure to Aβ, combined application of either Aβ and rosiglitazone or Aβ and FH535. (A) Representative traces of current clamp recordings indicating spontaneous firing activity of cultured pyramidal neurons for each experimental groups at 14 days *in-vitro*. (B) Exposure to Aβ alone and Aβ + FH535 led to a membrane depolarization compared to control and Aβ+ rosiglitazone. (C) Firing frequency was also affected by Aβ and combination treatment with either rosiglitazone or FH535. (D) Histograms of average amplitude of AP in different experimental conditions. ** and*** indicate *P *< 0.01 and *P *< 0.001 *versus *control neurons and ††† indicates *P *< 0.001 *versus *Aβ alone-treated neurons

The cultured neurons were divided into four groups (n > 50 neurons in each group): control, Aβ alone-treated, Aβ+rosiglitazone (ROS) and Aβ+FH535 treated. Aβ_1-42_ was prepared by dissolving the peptide in sterile PBS to a final concentration of 100 nM and incubated at 37 °C for 27 h. Rosiglitazone (30 µM) and FH535 (15 µM) were dissolved in DMSO. The final concentration of DMSO was 0.1% (v/v). At this concentration, it had no effect on neuronal activity (data not shown).After twelve days in culture, the neurons were exposed to either Aβ alone or in combination with rosiglitazone or FH535 for 24 h and then the whole-cell recordings were made as previously described (29). Briefly, a coverslip with cultured pyramidal neurons was placed in a recording chamber mounted on an inverted microscope (Olympus IX71, Japan) equipped with an Olympus DP12 camera and continuously perfused with HEPES-based artificial cerebrospinal fluid containing (in mM) 140 NaCl, 2 CaCl_2_, 1.4 KCl, 10 HEPES, 10 glucose, pH 7.3 (adjusted with NaOH) and osmolarity was 295-297 mOsml/L, bubbled with 100% O_2,_ and continuously flowed throughout the experiments at 2 mL/min. Cultured hippocampal pyramidal neurons were identified by their morphological characteristics (Figures 1A-1D). The micropipettes were pulled by a two-stage puller (PP-10, Narishige, Japan) from borosilicate glasses (1.5 O.D, 0.86 I.D.) with a tip resistance of 5-7 MΩ when filled with internal solution containing (in mM) 145 KCl, 3 NaCl, 0.5 CaCl_2_, 10 HEPES, 2 Na_2_ ATP, and 0.4 Na_2_ GTP. The pH of the internal solution was set to 7.3 by KOH, and the osmolarity was adjusted to 280-290 mOsm. 

All recordings were made at room temperature (22-25 °C) using a Multiclamp 700B amplifier (Axon Instruments, Foster City, CA). Data were filtered at 5 kHz and sampled at 10 kHz using a Digidata 1440 analog-to-digital convertor and pClamp 10 software (Axon Instruments, Foster City, CA) and stored on a personal computer for offline analysis. Recordings were discarded when the series resistance was over 20 MΩ. 

The resting membrane potential (RMP), the membrane resistance (R_in_), the firing frequency, and the after hyperpolarization (AHP) were measured under current clamp condition. The AHP amplitude was measured from the RMP to peak of AHP. Mean firing frequency was calculated as the number of action potentials over the 1 sec period. Rin was determined as the slope of the linear current-voltage relationship.

*Voltage-clamp experiments*

Inward Ca^2+^ currents were recorded with a pipette containing (in mM): 10 CsCl, 100 Cs2 MeSO4, 0.5 CaCl_2_, 10 HEPES, 0.5 EGTA, 2 Na_2_ ATP, 0.3 Na_2_ GTP, 5 QX-314, and, 20 TEA; pH 7.4 adjusted with CsOH, 295-297 mOsm/L. The extracellular ACSF solution for recording Ca^2+^ currents consisted of (in mM): 126 NaCl, 1.4 KCl, 2.5 CaCl_2_ , 10 Hepes, 10 Glucose , 3 Pyruvic acid and 2 mM 4-AP and 10 mM TEA were added to block outward K^+^ channel currents. The pH was adjusted with NaOH to 7.4 and osmalarity was 280-290 mOsmol/L. Ca^2+^ currents were elicited by voltage steps from -75 mV to +15 mV in 10 mV increments from a holding potential of -65 mV for 2500 msec. Furthermore, to assess the changes in the voltage sensitivity of Ca^2+^ channel current activation, the half maximal activation (V_half_) was determined by fitting individual conductance- voltage (G-V) relationship with a Boltzmann function using MATLAB (The MathWorks):

$\frac{G}{\mathrm{Gmax}}=1/\{1+\exp\left[\frac{-\mathrm{Vhalf}-\mathrm{Vm}}{k}\right]\}$

Where the G_max _is the mean value of maximal conductance, V_half _is the voltage for half-maximal activation of channels and k is the slope factor. The channel conductance was calculated according to the equation: 

 G = I_Ca_^2+ ^/(Vm-Vrev)

where G is the conductance, I_Ca_^2+ ^is the Ca^2+^ tail current, Vm is the holding voltage, and Vrev is the reversal potential of I_Ca_^2+^. V_rev _was determined to be about 100 mV by linear extrapolation to the peak of the tail current from clamping at voltages between -75 mV to 25 mV.

*Morphometric and electron-microscopic methods*

In order to characterize changes in cultured neuronal dimension the longest axis of the soma was measured. Eighty cultured pyramidal neurons were randomly chosen to measure their soma size (20 cell in each experimental groups). The ultrastructure of the cultured neurons was also evaluated at day 14 under electron microscope, as described by Bahrami *et al.* (30). Briefly, after two weeks, the cultured pyramidal neurons on plastic cover slips in different experimental conditions were fixed in 2.5% glutaraldehyde for 1 h with osmium tetroxide post-fixation for 30 min at 37 ^o^C then, rinsed with 3.6% NaCl and water. Thereafter, the cells were dehydration in graded ethanol and embedded in LR-white resin and after polymerization at 60 ^o^C for 3 days, the grids were made. Next, the sections were stained with uranyl acetate and lead citrate and observed with a Zeiss electron microscope.

*Assessment of cell viability*

Cell viability was performed as previously described using Cell Titre 96 Aqueous One Solution (MTS) Cell Proliferation Assay Kit (Promega Ltd.), which measures mitochondrial activity (29). Briefly, cultured neurons on coverslips either in control condition or following treatment with Aβ, Aβ +ROS, or Aβ + FH535 were washed with phosphate buffer saline (PBS). Then,100 µL of the serum-free culture medium containing 20 µL of mixture ofMTS[3-(4,5-dimethylthiazol-2yl)-5-(3-carboxymethoxyphenyl)-2(4-sulfophenyl)- 2H-tetrazolium] and PMS [phenazine methosulfate as the electron coupling reagent] was added into each well of the 96-well assay plates and re-incubated for 2 h at 37 ºC. The absorbance of the sample was recorded at 490 nm using a Wallac microplate reader. The MTS reduction values were expressed as percentage of the control (untreated cells). The percentage of viable cultured cells was then plotted as percentage of control.

*Statistical analysis*

The data were checked for normal distribution using a Kolmogorov–Smirnov test and then analysedusing one way ANOVA followed by Tukey post-hoc test.

The values were presented as mean ± SEM and significant difference between experimental groups was at *p* < 0.05.

## Results

The purpose of the present study was to examine the effects of PPARγ activation and Wnt signaling inactivation on Aβ-induced alterations in electrophysiological properties of the cultured hippocampal pyramidal neurons.

The results of the whole cell patch clamp recordings under current clamp condition indicated that hippocampal cultured pyramidal neurons exposed to Aβ alone exhibited lower spontaneous firing activity (Figure 1A). However, the combined treatment with Aβ and rosiglitazone preserved almost the normal spontaneous firing activity. Exposure of cultured neurons to Aβ plus FH535 resulted in spontaneous neuronal hyperactivity (Figure 1A). One-way ANOVA followed by Tukey’s post test revealed that treatment with Aβ alone had a significant effect on the resting membrane potential and caused depolarization of the cell membrane when compared either to control (-51.31 ± 0.59 mV *vs.* -48.67 ± 0.59 mV; *P* < 0. 01 ) or Aβ + ROS- treated group (-51.44 ± 0.35 mV; *P < *0.001, Figure 1B). In addition, co-treatment with Aβ plus FH535 (15 µM) led to a similar potential shift toward depolarization (-48.88 ± 0.43 mV; *P < *0.001; Figure 1B). However, co-exposure to Aβ plus rosiglitazone did not significantly affect the membrane potential compared to the control cells (Figure 1B).

To further investigate the impact of activation and inhibition of PPARγ/δ and possibly Wnt/β catenin pathway on neuronal excitability in the presence of neurotoxin Aβ, the spontaneous firing frequency was measured. Aβ treatment alone resulted in a significant reduction in the firing frequency of AP (from 2.16 ± 0.18 Hz in control group to 0.78 ± 0.16 Hz in Aβ treated neurons; *P < *0.001; Figure 1C). Wherase, combined treatment with Aβ and rosiglitazone preserved almost the normal neuronal firing compared to Aβ treatment alone group (1.9 ± 0.07 Hz; *P < *0.001; Figure 1C). On the other hand, application of Aβ plus FH535 induced neuronal hyperexcitability, as reflected by a significant increase in the firing frequency of the cultured pyramidal neurons compared to Aβ alone-treated group (3.88 ± 0.15 Hz; *P < *0.001; Figure 1C). 

**Figure 2:**
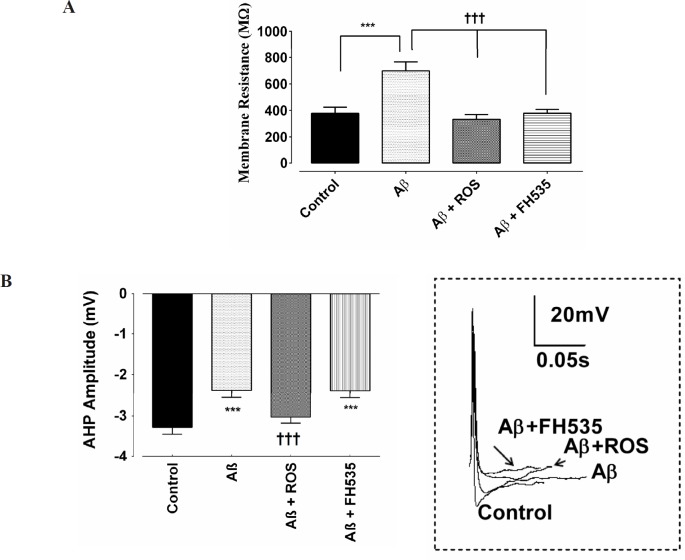
The impact of PPR-γ activation and inhibition on electrophysiological properties on cultured hippocampal pyramidal neurons

Furthermore, exposure to a low dose of Aβ led to a significant reduction in AP amplitude (from 64 ± 1.9 mV to 35 ± 0.92 mV; *P < *0.001). Although, the amplitude of action potential in Aβ+rosiglitazone treated group was statistically larger (54 ± 0.56 mV; *P < *0.001) than those recorded in Aβ-treated group, it was significantly smaller (*P < *0.001; Figure 1D) than the control group. Inhibition of PPAR-γ/δ by FH535 resulted in a significant (49 ± 1.4 mV; *P < *0.001) smaller and larger AP amplitude when compared to the control and Aβ-treated alone group, respectively. In addition, the cell membrane resistance was significantly increased in the presence of low concentration of Aβ (from 378.5 ± 21.9 MΩ in control to 705.8 ± 12 MΩ following Aβ treatment; *P < *0.001). Whereas, the application of Aβ in combination with either rosiglitazone (30 µM), or FH535 (15 µM) significantly decreased the membrane resistance, when compared to Aβ-treated alone (333.5 ± 13.63 MΩ; *P < *0.001; 369.5 ± 4.062 MΩ ; *P < *0.001, respectively; Figure 2A).

The AHP amplitude was also significantly reduced in Aβ-treated group when compared to the control neurons (-3.3 ± 0.17 mV in control, -2.39 ± 0.15 mV in Aβ, respectively; *P < *0.001; Figure 2B). Combined treatment of cultured pyramidal neurons with Aβ and rosiglitazone did not affect the amplitude of AHP when compared to the control value, but the AHP amplitude was significantly larger compared to Aβ-alone treated group (-3.03 ± 0.05 mV; *P < *0.001; Figure 2B). On the other hand, when FH535 was applied with Aβ, the amplitude of AHP was significantly lower than control (-2.40 ± 0.15 mV; *P < *0.001). 

**Figure 3:**
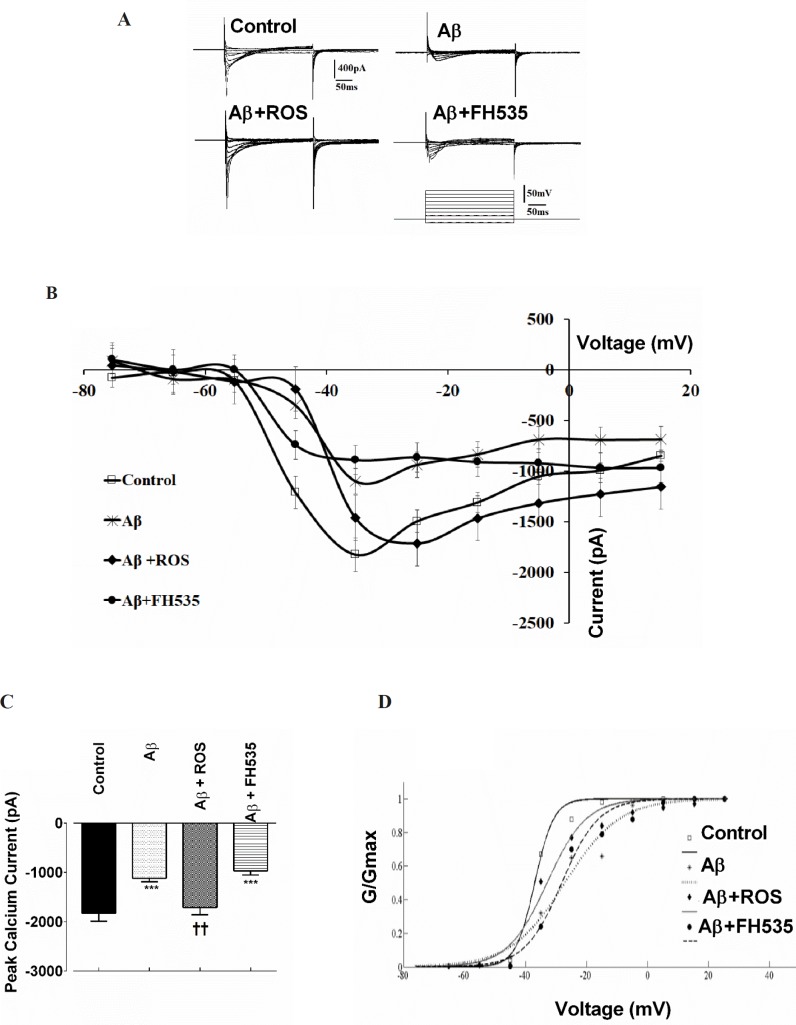
Effect of PPAR activation and inhibition on Aβ-induced alterations in Ca2+ channel currents. Representative macroscopic Ca2+ currents showing the effect of Aβ treatment alone or in combination with either a PPAR agonist or inhibitor. Currents were elicited by depolarizing voltage steps in 10 increments from -75 to 25 mV. Holding potential was -65 mV. (A) The pulse protocol is depicted at the bottom. (B) Current-voltage relationships and (C) average Ca2+ current peak amplitude for cultured pyramidal neurons in different conditions. (D) Normalized Ca2+ channel activation curves in control and following exposure to Aβ alone and after pre-treatments with either rosiglitazone or FH535. ***indicates *P < *0.001 *versus *control neurons and ††indicates *P < *0.01 *versus *Aβ alone-treated neurons

Next, to evaluate further whether Ca^2+^ channel inward currents may contribute to the altered electrophysiological properties of cultured pyramidal neurons caused by either Aβ alone-treated at low concentration or co-treatment of Aβ with rosiglitazoneor FH535, voltage clamp recordings were performed. Na^+^ inward and K^+^ outward currents were blocked by adding 5mM QX-314 to the Cs^+^-based intracellular solution and 4-AP (5 mM) to the ACSF, respectively. Following blockade of Na^+^ inward and K^+^ outward currents, membrane depolarization from -75 mV to +15 mV induced inward Ca^2+^ current in control neurons (Figures 3A and 3B) with a mean peak of -1828.48 ± 168.56 pA. Treatment with Aβ caused a significant decrease in the peak amplitude of the Ca^2+^ current to -1102.94 ± 51.81 pA, appeared at -35 mV (*P < *0.001; Figures 3B and 3C). The reduction of the inward current amplitude was observed over the entire voltage range tested (Figure 3B). The application of rosiglitazone in combination with Aβ induced an inward Ca^2+^ current that reached to peak amplitude of -1715 ± 198.45 pA at -25 mV which was significantly higher than the value obtained from Aβ alone -treated groups (*P < *0.01; Figure 3B and 3C). 

The current-voltage relationship showed that the Ca^2+^ current in Aβ+FH535 treated cultured neurons was significantly lower (-969.70 ± 81.77 pA; *P < *0.001) than the control but not than that of Aβ alone- treated neurons (Figures 3B and 3C).

In addition, normalized Ca^2+^ channel activation curves in groups with either Aβ treatment alone or in combination with FH535 showed a non significant shift in the midpoint activation toward depolarized potentials (26.23 ± 6.76 mV in Aβ-treated and 28.62 ± 0.94 mV in Aβ + FH535 treated group, *P* > 0.05, 10 cells in each group; Figure 3D) when compared to control (32.95 ± 0.16 mV) or Aβ + FH535 (33.87 ± 2.48 mV). The slope factors were not also significantly altered (data not shown). 

**Figure 4:**
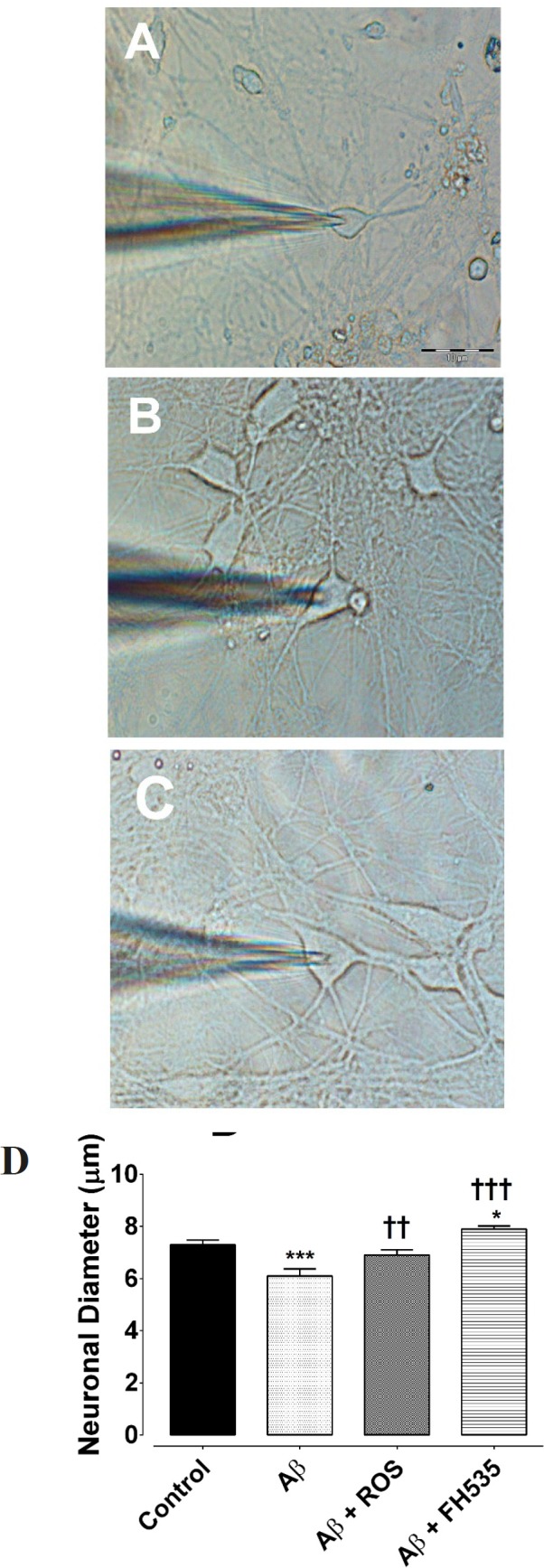
Hippocampal pyramidal neurons cultured in Neurobasal/B27 medium. (A) Primary cultured neurons were photographed at DIV 14 in control, (B) following 24 h exposure to Aβ, (C) after treatment with either Aβ + rosiglitazone or (D) Aβ + FH535 for 24 h. The histogram indicates the neuronal diameter in different conditions. Exposure to Aβ led to a significant decrease in neuronal soma size. Activation of PPR-γ by rosiglitazone preserved the cell soma size in the presence of Aβ, but exposure to FH535 did not prevent the effect of Aβ on cell soma size. *indicates *P < *0.05, ***indicates *P < *0.001 *versus *control neurons, †† and ††† indicate *P < *0.01, *P <*0.001 *versus *Aβ alone-treated neurons

Furthermore, light and electron microscopic examination of hippocampal pyramidal cultured neurons at day 14 showed that exposure to 100 nM Aβ (1-42) reduced neuronal diameter (from 7.3 ± 0.1 µm in control condition to 6.1 ± 0.9 µm flowing Aβ treatment, *P < *0.001, n = 20; Figures 4A and 4B). However, Aβ when applied in combination with either rosiglitazone (6.9.08 ± 0.27 µm, *P < *0.01, n = 20) or FH535 (7.899.08 ± 0.24 µm, *P < *0. 001, n = 20; Figures 4A and 4B) not only did not induce a reduction in soma size, but also significantly increased the soma diameter. 

(A) Effects of activation and inhibition of PPR-γ and possibly Wnt/β signaling pathway on the changes in input resistance and (B) AHP amplitude induced by Aβ alone and in combination with rosiglitazone or FH535. Inset: Superimposed AHPs from neurons in different experimental conditions. *** and ††† represent *P < *0.001 *versus *control and Aβ treatment alone.

**Figure 5:**
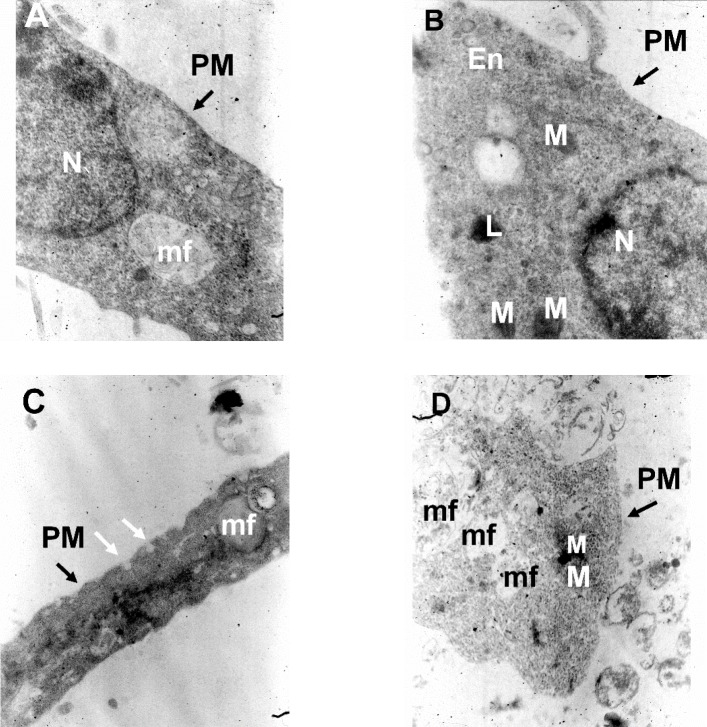
TEM images of cultured hippocampal pyramidal neurons in different experimental conditions. (A) Electron micrographs of a neuronal soma in the control condition, (B) following treatment with Aβ alone, (C) following Aβ plus rosiglitazone and (D) Aβ plus FH535. Images are from a 14 days old culture. N: Nucleus; M: Mitochondria; PM: Plasma Membrane; L: lysosome; mf: myelin figure; ER: Endoplasmic reticulum; En: Endosome vesicles; White arrows indicate endocytotic membrane invagination. 20000X magnification

**Figure 6:**
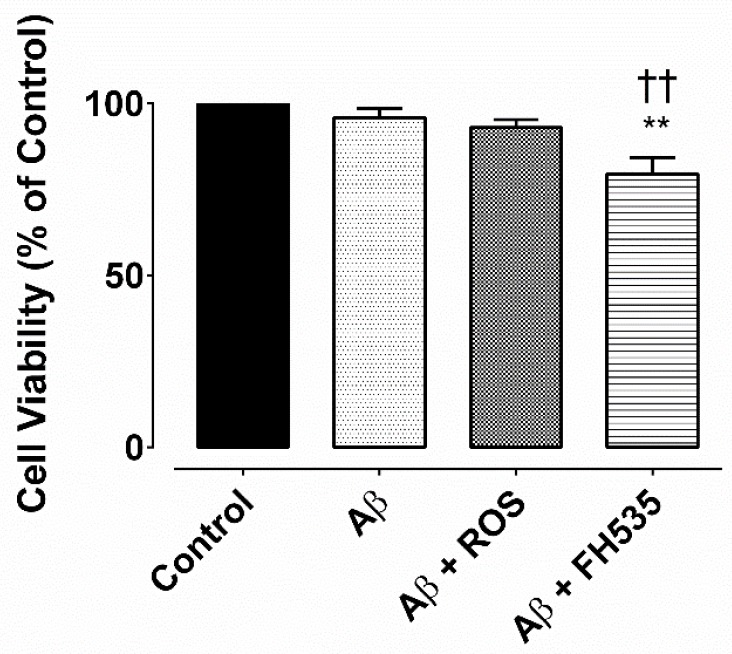
MTS assay for cell viability. Cell viability was determined by MTS assay and it was expressed as a percentage of controlExposure to Aβ alone or Aβ plus rosiglitazone did not reduce the cell viability, but after combine treatment with Aβ and FH535 reduced the cell viability. ** and †† indicate *P< *0.01 *versus *control neurons and Aβ alone-treated neurons,respectively

The results of the electron microscopic examination indicated that low concentration of Aβ treatment alone did not induce any ultrastructural changes indicative of any signs of neuronal degeneration or damage.

The plasma membrane integrity was not affected by either treatments (Figure 5). In control and Aβ-treated groups, nuclei were large and round or oval with homogeneous karyoplasm (Figures 5A and 5B). The cytoplasms were homogenous and contain mitochondria and lysosome (Figure 5B). The nucleus was not observed in the sections obtained from either Aβ+ROS or Aβ + FH535 treated groups (Figures 5C and 5D). The Aβ+ROS treated cultured pyramidal neurons showed dome-shaped endocytic membrane invaginations at the cell surface (Figure 5C). In addition, myelin figures were also observed which were more evident in Aβ+FH535 treated cultured neurons (Figure 5D).

Myelin figures, fingerprint or onion multi-laminated -like structures originated possibly from breakdown of membrane and organelles, composed of osmophilic materials driven from damaged membranes (*e.g.* endoplasmic reticulum) and they are considered as a sign of degeneration of nerve processes (32, 33).

The MTS assay indicated that there was no significant difference between the cell viability of the untreated cells (control) and bothAβ (100 nM) treated and Aβ + rosiglitazone; however, exposure to combined treatment with Aβ and FH535 significantly reduced the cell viability (*P < *0.01) as compared to either to control or Aβ-treated cultured neurons (Figure 6).

## Discussion

Deregulation of Wnt signaling has been proposed as an ethiological cause of AD (34); therefore activation of Wnt signaling pathway has been shown to rescue fibrillar Aβ-induced neurodegeneration (35). There are evidences indicating that accumulation and excessive concentration of Aβ substantially increases the risk of AD in which synaptic dysfunction and ultimately neuronal cell loss occurs (36, 37). However, very few studies have focused on the functional impairments at the beginning of the disease in response to neuronal exposure to the low concentration of Aβ and still the mechanism behind the action is not fully understood (38-41). On the other hand, several research studies have been conducted on a number of potential compounds that target the Aβ for the treatment of AD (41-43, 45). In this context, PPAR-γ agonist could be considered as a treatment option for AD*.* There are abundant evidences to support the beneficial effects of PPAR*-γ* activation against neurodegenerative diseases, including AD (45-48); however the electrophysiological mechanisms by which PPAR-γ activation prevents Aβ-induced functional alterations and the possibility of the use of its agonists at the early stages of AD have not been fully elucidated. In the current study, it was hypothesized that PPAR-γ and Wnt signaling pathway may affect the functional changes induced by low concentration of Aβ (100 nM). Thus, it was attempted to determine the electrophysiological consequences of the effect of rosiglitazone and FH535, which are generally accepted as an agonist and antagonist of PPAR-γ and Wnt signaling (31, 41 and 52), against functional alterations induced by Aβ. 

Recent lines of evidences have indicated that Wnt signaling may be involved in synaptic plasticity, modulation of long-term potentiation and in neuroprotection against Aβ (20, 53 and 55). However, little detailed electrophysiological information, if any, is available concerning the effect of Wnt signaling on neuronal excitability and action potential characteristics. Here, our findings revealed that exposure to a low concentration of Aβ (100 nM) for 24 h altered the electrophysiological properties of cultured hippocampal pyramidal neurons as evidenced by a significant reduction in the firing frequency accompanied by a decrease in Ca^2+^ channel current amplitude, without a significant alteration in cell viability and ultrastructural features of the cultured neurons. Several studies have reported the deleterious effect of Aβ exposure at low concentration on cell function (55) without causing cell death (41). The present results are also consistent with previous reports, which have demonstrated the damping effect of Aβ (1-42) on neuronal excitability in animal models of AD and impairment of hippocampal long-term potentiation (39, 56-59). Furthermore, nanomolar doses of Aβ obtained from the human cortex of AD patients have been shown to cause neuronal alterations (60). Aβ (1-42) at low concentration has also been shown that suppresses the spontaneous synaptic activity by inhibition of Ca^2+^ channel currents, which is in agreement with our finding on Ca^2+^ channel currents in cultured pyramidal neurons (55). However, there is evidence showing that increasing Aβ accumulation and toxicity generates an increment in the intracellular Ca^2+^ by forming pore or channels permeable to Ca^2+ ^and thereby causes cell death (37, 61 and 62). This inconsistency might be due to the low concentration Aβ that was used in the present study.

Based on the above evidences and having this in mind that neuronal exposure to low concentration of Aβ disrupts the electrical functionality of neurons before affecting the cell viability and ultrastructural features, which may occur at the early stage of AD. Then, we evaluated the protective effect of PPAR-γ and possibly Wnt signaling pathway against electrophysiological consequences of Aβ exposure. Cultured neurons retained their almost normal electrophysiological properties following treatment with combined treatment with a PPAR-γ and Wnt signaling pathway activator, rosiglitazone (30 µM), and Aβ (100 nM). 

The potential protective and beneficial effects of activation of PPAR-γ (or Wnt signaling pathway) have been assessed in animal models of AD (51, 63 and 65). Disruption of Wnt signaling pathway has been found to play a role in Aβ-induced neurotoxicity and activation of this signaling cascade exerts the preventive effect on such cytotoxic effects (14, 15 and 51); however, the mechanism of the effect is not fully elucidated yet. Here, exposure to Aβ together with rosiglitazone resulted in almost normal electrophysiological firing behavior in cultured pyramidal neurons when compared to control neurons. This combined treatment was also diminished the inhibitory effect of Aβ treatment alone on Ca^2+^ channel current. The increased neuronal excitability induced by co-treatment of Aβ and rosiglitazone could be possibly due to a significant increase in the AHP amplitude, which in turn determines the availability of Na^+^ channels by speeding their recovery from inactivation (65, 66). In CA1 pyramidal neurons of a mouse model of AD has been found that overproduction of Aβ altered neuronal excitability and reduced Na^+^ current (67). In addition, reduction in the amplitude of AP could also be consistent with the hypothesis that Aβ might inhibit the Na^+^ channels function.

It has been reported that pre-treatment with PPAR-γ antagonists reversed the protective effect of PPAR-γ activation on memory impairment (68). Therefore, the impact of inhibition of PPAR-γ and δ using FH535, which has been shown to be an inhibitor of both Wnt and PPAR signaling, was assessed in the presence of low concentration of Aβ (31, 52). Cultured neurons exposed to FH535 plus Aβ showed significant lower cell viability when compared to either control or Aβ alone-treated groups and the inhibitory action of FH535 preserved the disruptive effects of Aβ on electrophysiological properties of cultured neurons. Co-exposure to FH535 and Aβ was associated with a decrease in AHP and an increase in AP firing rate and there by induced neuronal hyperexcitability. Application of FH535 preserved the inhibitory effect of Aβ on Ca^2+^ channels current, when co-treated with Aβ. One possible explanation for the observed hyperexcitability following exposure to Aβ+FH535 could be the attenuating effect of combined treatment of Aβ with FH535 on K^+^ outward current (*e.g. *Ca^2+^ dependent K^+^ outward current). 

It has been very recently demonstrated that activation of PPAR/γ by agonist caused stimulation of BKCa^2+^ channels in hippocampal neurons (69). Voltage-dependent and calcium-activated potassium channels are critical players in opposing hyperexcitability in neurons and thereby play a crucial role in regulating the excitability and firing behavior of neurons (70, 71). However, this needs further examination and direct confirmation by use of voltage-clamp recording.

Exposure to low concentration of Aβ was associated with an increase in the membrane resistance and a decrease in the soma size, but cotreatment with rosiglitazone resuced both the neuronal soma size and the normal membrane resistance. An inverse correlation between neuronal soma size and membrane input resistance has been reported (72-74). The increase in the membrane input resistance could be due in part to the decrease in the hyperpolarization-activated non selective cataionic current (75, 76). However, othe factors including a reduction in the leak channel due to the cell shrinkage could be also involved. On the other hand, application of FH535 led to a decrease and an increase in the membrane resistance and soma size, respectively. The leak channels determine the membrane input resistance and opening of these channels may induce cell swelling and thereby can cause hyperexcitability and neuronal death (77-79). Although, we have not provided proof for the possible effect of FH535, an inhibitor of Wnt signaling, on leak channels, there are reports that Wnt signaling regulates hippocampal neurogenesis and promot neuronal survival, as in Wnt knockout mice the neuronal loss has been shown (80-81).

## Conclusion

In conclusion, this study demonstrated that Aβ at low concentration caused electrophysiological alterations which may lead to functional neuronal impairment without affecting the cell survival and cell structure. Activation of PPAR-γ and Wnt signaling could be a therapeutic target for the treatment of AD at the earlier stages of the disease before a detectable sign of cell death.
